# Preoperative positive urine nitrite and albumin-globulin ratio are independent risk factors for predicting postoperative fever after retrograde Intrarenal surgery based on a retrospective cohort

**DOI:** 10.1186/s12894-020-00620-7

**Published:** 2020-05-06

**Authors:** Zhong-yu Jian, Yu-cheng Ma, Ran Liu, Hong Li, Kunjie Wang

**Affiliations:** 1 Department of Urology, Institute of Urology (Laboratory of Reconstructive Urology), West China Hospital, Sichuan University, No. 37 Guo Xue Xiang, Chengdu, Sichuan 610041 P.R. China; 2 West China Hospital, Sichuan University, Chengdu, P.R. China

**Keywords:** Fever, Risk factor, Urine nitrite, Albumin-globulin ratio, Retrograde intrarenal surgery, Nomogram

## Abstract

**Background:**

To determine risk factors for postoperative fever (POF) after retrograding intrarenal surgery (RIRS) and a nomogram for prediction of POF in patients undertaking RIRS has been developed based on the risk factors found.

**Methods:**

This is a retrospective designed-study. A continuous cohort from a single-center database that consisted of 1095 cases undertaking RIRS with complete preoperative medical records from January 2009 to December 2018 was obtained. Independent risk factors were identified according to the multi-variate logistics regression and a further nomogram was developed. The performance of the nomogram was evaluated through three aspects including net clinical benefit, calibration, and discrimination.

**Results:**

A total of 31(2.8%) cases had POF after the RIRS. Risk factors included time in RIRS ≥30mins (only the flexible scope use period) (OR: 2.16, 95%CI; 1.01–4.62, *P* = 0.047), preoperative positive urine culture (OR: 2.55, 95%CI; 1.01–6.42, P = 0.047), preoperative positive urine nitrite (OR: 9.09, 95%CI; 2.99–27.64, *P* < 0.001), Albumin/globulin ratio (AGR) (OR: 0.14, 95%CI; 0.03–0.74, *P* = 0.020) were further included in the nomogram to predict the POF probability for individuals. The Hosmer-Lemeshow test showed a goodness-of-fit. The calibration curve demonstrated good agreement between observation and prediction. Decision curve analysis (DCA) demonstrated it was clinical use in RIRS.

**Conclusions:**

The preoperative urine nitrite, AGR, RIRS time, and preoperative urine culture are found to be independent risk factors associated with POF after RIRS. Then we have developed a nomogram taking these factors into account that accurately predicted POF after RIRS.

## Background

Over the last decades, the surgical treatment of kidney stones has changed with the development of technology. The use of ureteroscopy (URS) increases and has become the most common choice over time [1]. According to previous reports, the incidence of postoperative fever (POF) after ureteroscopic holmium laser lithotripsy is 2–28% [2]. POF is potentially serious because it may progress to urosepsis which leads to mortality [3]. Therefore, identifying high-risk POF individuals and strengthening the interventions are urgently needed.

Several studies reported the risk factors for POF after URS, and many of them focused on retrograde intrarenal surgery (RIRS) [4–6], both were limited by the small sizes and the number of potential risk factors included. Besides, this question was not fully answered. Therefore, this paper aimed to determine risk factors for POF after RIRS. Additionally, we also developed a nomogram for predicting the risk of POF.

## Methods

### Study population

From January 2009 to December 2018, patients undertaking RIRS in our region by a single surgeon (WK) were screened after local health ethics approval in west china hospital was granted for our study. Patients with incomplete information about the risk factors described below were excluded. Different RIRS operations of the same patient were counted independently. A total of 1095 cases of RIRS were successfully collected and analyzed.

### Outcomes and predictors

POF information was defined as temperature ≥ 38 °C following RIRS 72 h after operation [6, 7]. Predictors in our study were mainly classified into three categories based on the literature review. 1. Demographic characteristics and medical history included age, gender, smoker, alcoholics, body mass index (BMI), diabetes, hypertension, chronic kidney disease, Coronary heart disease, hyperuricemia, hepatitis history, tuberculosis history, allergy history, blood transfusion history, extracorporeal shock wave lithotripsy (ESWL) history, residential zone, anticoagulant treatment, RIRS history, Percutaneous nephrolithotripsy (PCNL) history, incision lithotomy history, Charlson comorbidity index. 2. Pre- and intraoperative characteristics included preoperative ureteral stent indwelling time, access sheath size, time in RIRS and time in extracting stone fragments. Imaging information included the number of stones, stone location, stone size and computerized tomography (CT) value of stones. 3. Laboratory tests mainly focused on the indexes which can be used to asses preoperative urinary tract infection situation, immunity function, nutrition situation and coagulating functions (supplementary Table 1).

### Preoperative procedures and surgical technique

The details were described in our previous study [8]. Since preoperative double-J stent insertion could significantly increase the clear stone rate, in our institute the ureteral stent was generally placed approximately 2 weeks before surgery [9]. One or two days before the retrograde intrarenal surgery, blood laboratory test, urine laboratory test, and urine culture were routinely performed for all patients. Prophylaxis with a single dose of antibiotics 1 day before surgery was carried out if patients did not have infectious symptoms and had a negative urine culture result (usually Cefidipine). Otherwise, antibiotic treatment was used until both urine culture and urine laboratory tests became negative according to the antibiotic sensitivity test. Antibiotics are routinely used intraoperatively and postoperatively, and the antibiotics used are generally the same as those used before surgery. During the operation, we also strictly controlled the duration of the operation and the irrigation pressure. As in other reported cases [10], the use of antibiotics exceeded the American Urological Association (AUA) guideline which recommended no need use of antibiotics for asymptomatic patients before surgery [11].

Dexamethasone and furosemide were used at the beginning of surgery by the anesthetist. The access sheath was routinely used. The same standard (not same one) flexible ureteroscopes (Olympus-P5 5.3Fr) was advanced through the working sheath (14/16 Fr or 12/14 Fr) and the laser lithotripsy was performed. After the lithotripsy, the ureteral stent was indwelled for about 2 weeks.

### Statistical analysis

Continuous and categorical parameters were presented as the median (interquartile range [IQR]) and number (percentage) respectively. Variables found to be significant (*P* < 0.1) through univariable analysis were included in the further multivariable logistic regression analysis. If there is any possibility of insufficient sample size, the bootstrap resampling test should be applied to check significance. In any bootstrap regression stage, 10,000 times resampling was performed to ensure accuracy.

Based on the risk factor identified in multivariate analysis, a nomogram was built. Hosmer-Lemeshow test was performed to check the model’s goodness-of-fit and a calibration curve was also plotted [12, 13]. The 10-fold test for internal validation was performed following the model construction to check the performance of the nomogram. To guarantee the accuracy, 10-fold verifications were repeated 10,00 times to calculate the average area under the curve (AUC) for predicting the model. Also, the net benefit ratio was evaluated by decision curve analysis (DCA) [14].

All statistical analyses in our study were performed with R v.3.5.3 (www.r-project.org). *P* values mentioned in this paper were calculated in two-sides, with *P* < 0.05 was considered significant.

## Results

31 (2.8%) of 1095 cases had POF after the RIRS. Characteristics of included predictors and univariable logistic regression analysis results were displayed in supplementary Table 1. Then multivariate logistic regression analysis was performed enrolling these significant variables (*P* < 0.1). After the multivariable logistics regression and bootstrap resampling tests, time in RIRS ≥30mins (only the flexible scope use period) (OR: 2.16, adjusted 95%CI:1.01–4.62, adjusted *P* = 0.047), Preoperative positive urine culture (OR: 2.55, adjusted 95%CI: 1.01–6.42, adjusted P = 0.047), Preoperative positive urine nitrite (OR: 9.09, adjusted 95%CI: 2.99–27.64, adjusted *P* < 0.001), Albumin/globulin ratio (AGR) (OR: 0.14, adjusted 95%CI: 0.03–0.74, *P* = 0.020) were statistical significant and were regarded as independent risk factors for predicting POF (Table 1).

**Table 1:** Independent risk factors of POF based on multivariable logistics regression analysis

Variables	OR	95%CI^*^	***P*** value^*^
Time in RIRS ≥30mins	2.16	(1.02, 4.62)	0.047
Preoperative urine culture (positive)	2.55	(1.01, 6.42)	0.047
Preoperative positive urine nitrite (positive)	9.09	(2.99, 27.64)	< 0.001
Blood albumin/globulin ratio	0.14	(0.03, 0.74)	0.02

^a^Value was adjusted by 10,000 times bootstrap resampling

*POF* Postoperative fever, *RIRS* Retrograde intrarenal surgery, *ESWL* Flexible ureteroscopic lithotripsy, *OR* Odds ratio, *CI* Confidence interval

**Fig. 1:**
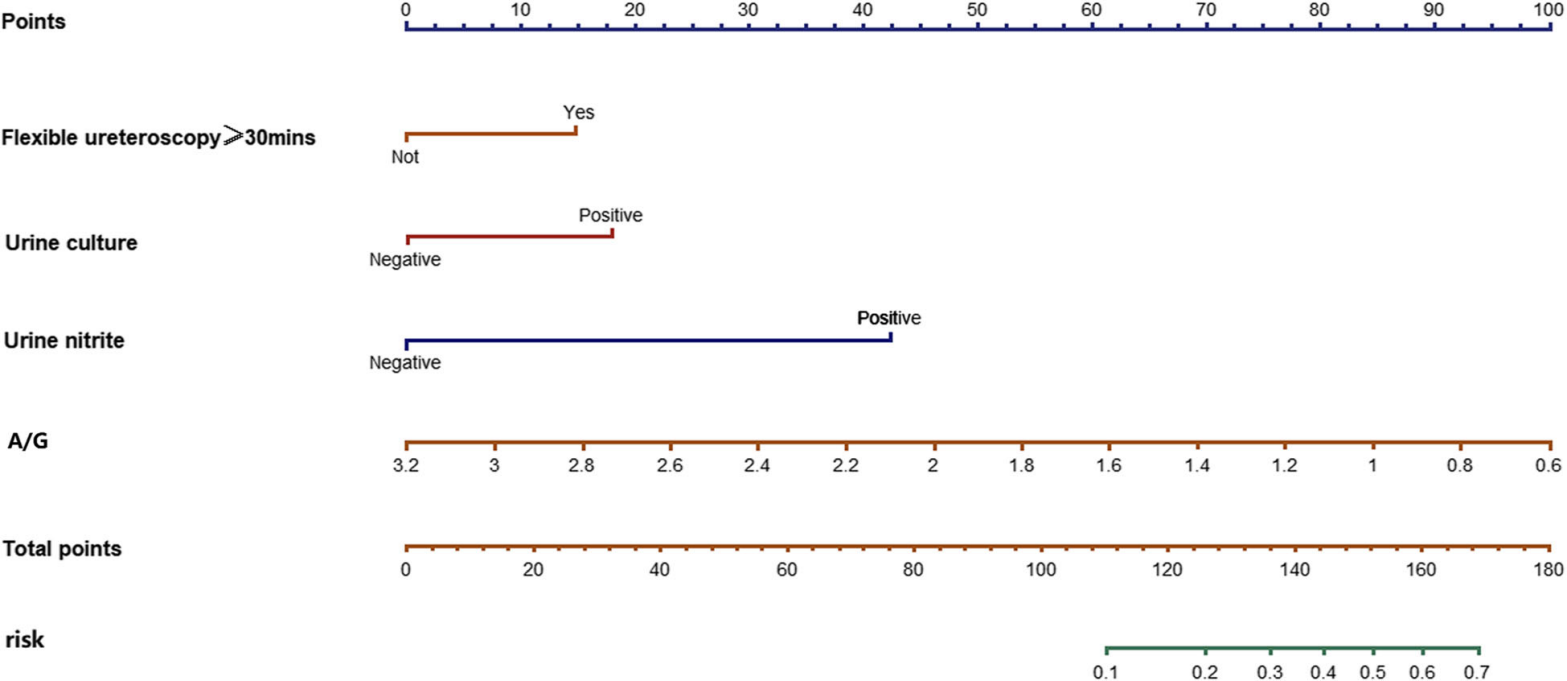
Nomogram for predicting POF after RIRS. Locate each variable on the corresponding axis and obtain a single score. Adding these single scores get the final total points. Draw a line straight up to find the probability of POF

**Fig. 2:**
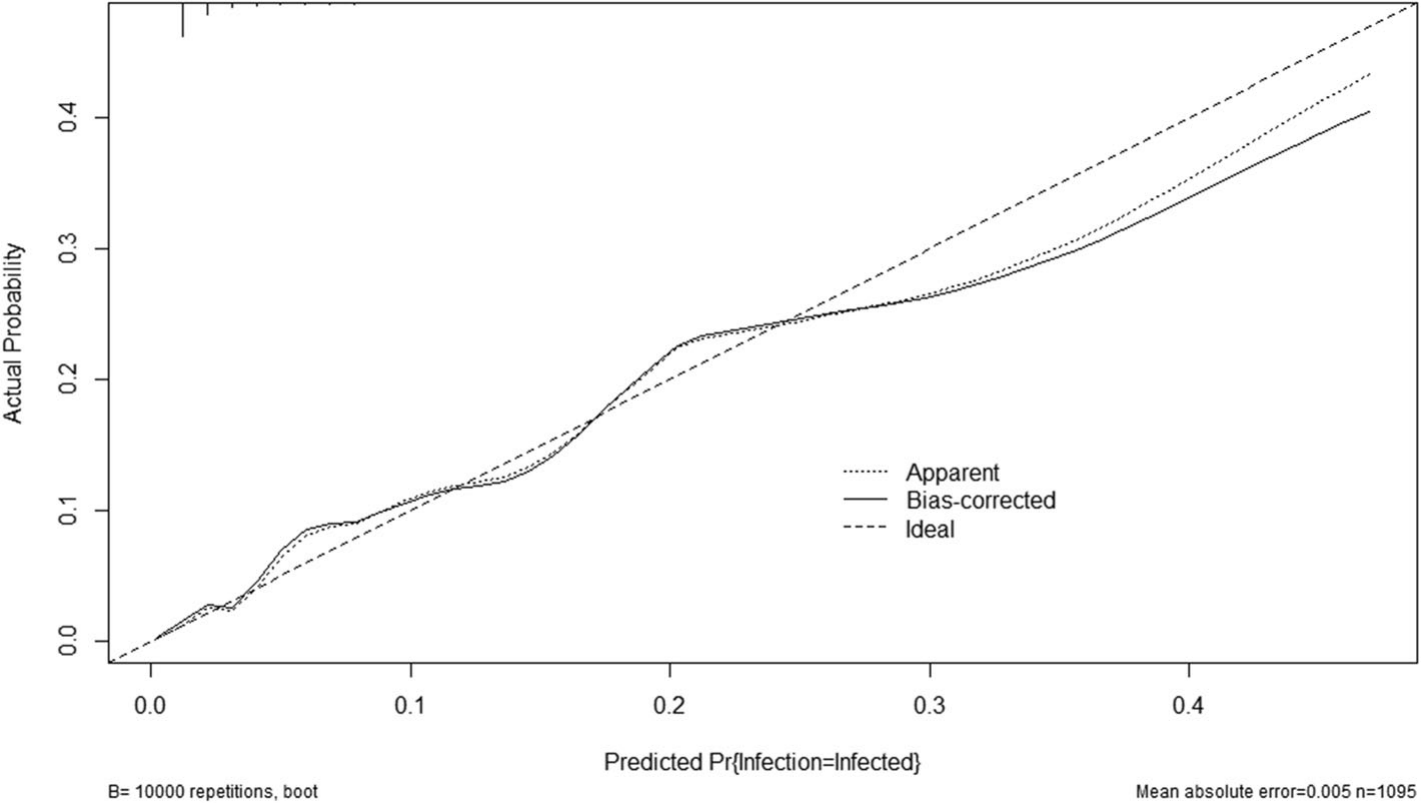
Calibration curves of the nomogram. The y-axis represents the actual POF rate. The x-axis represents the predicted POF risk

**Fig. 3:**
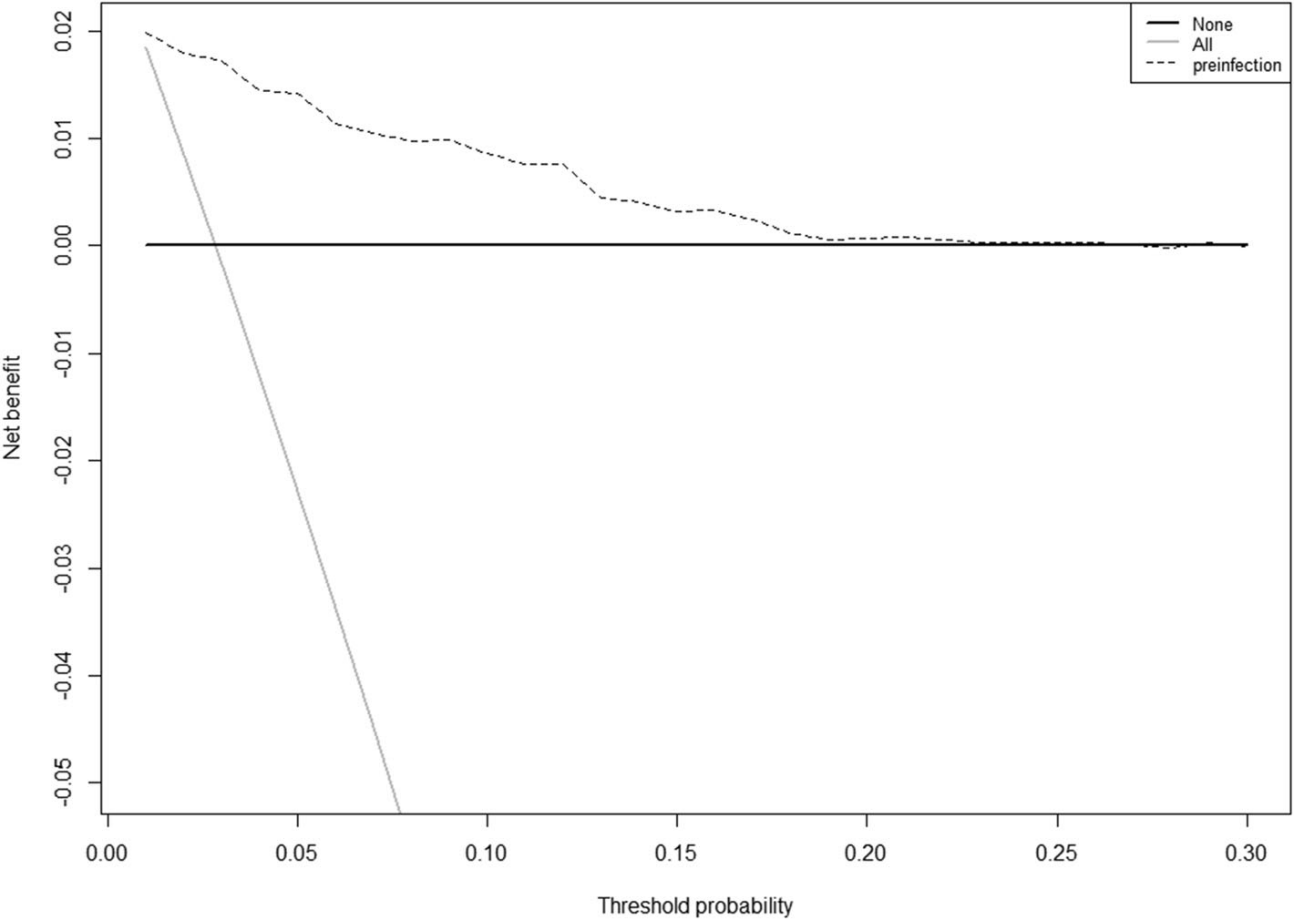
Decision curve analysis for the nomogram. The decision curve showed that nomogram conferred a greater clinical benefit with threshold POF probabilities at 1 to 20%

A nomogram based on multivariable logistic regression was built (Fig. 1). After 1000 times 10-fold internal validation, the average AUC was 0.835 with 95% CI (0.816, 0.870). There was no significant statistic in the Hosmer-Lemeshow test (Chi-Square = 8.29, DF = 8, *P* = 0.4056), which indicated that this nomogram had good goodness-of-fit. The calibration curve was shown in Fig. 2 and it demonstrated good agreement between observation and prediction in our POF predicting model. DCA indicated that the application of the POF predicting model could bring clinical net benefit when the probability is 1 to 27%, whereas the analysis does not detect any clinical net benefit after this threshold (27%) (Fig. 3).

## Discussion

URS including RIRS has become the most performed surgery in ureteral and kidney stone disease therapy [1]. Though the postoperative infectious complications rate is rare [15], they are potentially serious especially the occurrence of urosepsis. However, despite the risk factors reported by several studies, the question of predicting POF was not fully answered. At present, a single dose of antimicrobial prophylaxis 1 hour before surgery is recommended by AUA best practice statement [16]. However, the extended prophylaxis and adjusted postoperative use of antibiotics can be recommended for those individuals who are at high risk of POF. Thus, we tried to demonstrate independent risk factors for the POF and developed a nomogram. The probability derived from the nomogram could help identify high-risk patients.

The most significant predictor from our study was preoperative positive urine nitrite. Besides, to our knowledge, this was the first study reporting that preoperative positive urine nitrite was an independent risk factor for POF after URS, RIRS or PCNL. Nitrite is not normally present in urine unless uropathogens metabolite urinary nitrate. It can be detected if there are recordable and stable concentrations produced by bacteria incubated in the urine. A meta-analysis showed that positive nitrite can indicate urinary tract infection (UTI) with a probability of 99% in children of any age [17]. Similar high sensitivity was also reported in the studies enrolling elderly patients. More importantly, canceling urine cultures in negative leukocyte esterase and nitrite patients could reduce urine cultures by 40% and decreased inappropriate antibiotic use without compromise safety [18–20]. Also, in the elder hospitalized patients, the diagnosis of a UTI through the presence of bacteriuria in urine culture should be treated with caution because the prevalence of asymptomatic bacteriuria is so high (15–35% in men and 25 to 50% in women) [18]. Therefore, urine nitrite might play a pivotal role in UTI. Unlike urinary culture, urine routine test containing nitrite index is standard practice before RIRS in our center and can obtain results quickly. Demonstrating this risk factor (preoperative positive urine nitrite) can help to decide whether to use antibiotics before the outcome of urine culture. Besides, it can also help to understand if it is necessary to undertake urine culture among patients with preoperative negative urine nitrite. Of course, this requires prospective randomized control trials (RCTs) to further explored.

Paralleling the literature, preoperative positive urine culture was found to be an independent risk factor associated with POF [6, 21–23]. It was also identified as risk factors for infectious complications after PCNL [24]. In our clinical practice, urine cultures were standard obtained. Prophylaxis with a single dose of antibiotics 1 day before surgery was carried out if patients did not have infectious symptoms and negative urine culture. Otherwise, antibiotic treatment was used. Like the report by Southern et al. [6], we also recognize this exceeded the AUA guidelines which recommend that asymptomatic individuals did not need antibiotic therapy before surgery [11]. However, we thought this stratagem also contributed to the low rate of POF (2.8%).

Longer RIRS time (only the flexible scope period of use) was in our series, another risk factor independently associated with POF after RIRS. In RIRS surgery, longer operative time may not only mean a more complicated operation due to the stone burden but also can strengthen the effect of intrarenal pressure. Several previous studies also showed longer operation time was associated with postoperative infectious complications [5, 6]. Some predictors were not statistically significant might because they contribute to longer operation time together.

Additionally, AGR was found to be an independent protective factor for POF in our cohort. AGR was reported as a simple and valuable factor predicting the prognosis of several diseases, such as lung cancer [25], nasopharyngeal cancer [26], breast cancer [27], and so on. We assumed that albumin negatively affected the POF rate mainly in two ways. On the one hand, albumin is commonly considered as a marker to reflect nutritional status. And the nutritional status is associated with the immune response. Studies had reported that nutritional deficiency status (low albumin level) was associated with the frequency and severity of infection [28, 29]. On the other hand, albumin is likely to mobilize polyunsaturated fatty acids (PUFA) and aid in the formation of several anti-inflammatory lipids [30]. Therefore, low blood albumin levels may tend to pro-inflammatory status. In conclusion, albumin was expected to be negatively interrelated with both nutrition and inflammation, which partially contributed to the POF after RIRS.

After identifying independent risk factors for POF, we aimed to develop a nomogram. Nomogram helped us calculate how many points toward the probability of POF for each risk factor. Though this was not the first nomogram for predicting POF after RIRS, the previous study only enrolled 337 patients [5]. The DCA and excellent calibration in our study indicated that our model could confer net clinical benefit with threshold probabilities at 1–20%. And the POF rate reported in most studies was below 20% [2]. Thus, the nomogram which incorporated these four risk factors could serve as a convenient model for predicting POF among those individuals who undertook RIRS.

The retrospective design in our study was viewed as the main limitation, some valuable variables such as intrapelvic pressure could not be discussed in this study since almost all patients with RIRS received low flow irrigation with about 90 ml/min but it is still an issue worth discussing in the future studies. Our electronic medical records system helps us to track patients for an emergency visit, as well as readmission through other urologists, so it is less likely to lose POF patients based on our definition. Besides, though the patient loss rate met the requirements of statistical power, losing patients due to no record of surgery time was another limitation. Furthermore, we did not test the performance of previously published nomogram (337 patients) because the core independent risk factor C-reacting protein (CRP) in their study is not our routine tested index in our medical region. AGR and urine nitrite are two kinds of laboratory indicators that can be easily obtained in most institutions. Lastly, further large prospective e multicenter studies were needed to evaluate the validity of our model especially urine nitrite and AGR for the prediction of POF after RIRS.

## Conclusion

In conclusion, the preoperative urine nitrite, AGR, RIRS time, and preoperative urine culture are found to be independent risk factors associated with POF after RIRS. Then we have developed a nomogram taking these factors into account that accurately predicted POF after RIRS. This model may be conveniently used to identify patients at high risk for POF after RIRS. It can guide both preoperative counseling and scheduling preoperative antibiotic use.

## Supplementary information

**Additional file 1: Table S1**. Characteristics of included predictors and univariable logistic regression analysis results

## Data Availability

The datasets generated and/or analyzed during the current study are not publicly available due to local regulations on the management of medical records but are available from the corresponding author on reasonable request.

## Abbreviations

POF: Postoperative fever
RIRS: Retrograde intrarenal surgery
OR: Odds ratio
DCA: Decision curve analysis
AGR: Albumin/globulin ratio
URS: Ureteroscopy
BMI: Body mass index
ESWL: Extracorporeal shock wave lithotripsy
PCNL: Percutaneous nephrolithotripsy
CT: Computerized tomography
IQR: Interquartile range
AUC: Area under curve
DF: Degree of freedom
AUA: American Urological Association
PUFA: Polyunsaturated fatty acids
CRP: C-Reacting protein
