# Supplementary material for: Preoperative positive urine nitrite and albumin-globulin ratio are independent risk factors for predicting postoperative fever after retrograde Intrarenal surgery based on a retrospective cohort
Source: BMC Urol. 2020 May 6;20:50. doi: 10.1186/s12894-020-00620-7 (PMC7201725; doi:10.1186/s12894-020-00620-7)
Supplement: Supplementary file 1 — Additional file 1: Table S1. Characteristics of included predictors and univariable logistic regression analysis results [file 12894_2020_620_MOESM1_ESM.docx]

Table S1. Characteristics of included predictors and univariable logistic regression analysis results

| Predictors | N (%) or median (IQR) | P value^*^ |
| --- | --- | --- |
| *Demographic characteristics and medical history* |  |  |
| Gender |  | ***0.06*** |
| Male (n, %) | 739(67.5) |  |
| Female (n, %) | 356(32.5) |  |
| Age (years) | 48(39, 6) | 0.164 |
| BMI (kg/m^2^) | 24(21.9, 26.3) | 0.968 |
| Smoker (n, %) | 360(32.9) | 0.941 |
| Alcoholics (n, %) | 297 (27.1) | 0.865 |
| Diabetes (n, %) | 70(6.4) | 0.989 |
| Hypertension (n, %) | 176(16.1) | 0.993 |
| Chronic kidney disease (n, %) | 6(0.5) | 0.999 |
| Hyperuricemia (n, %) | 12(1.1) | 0.999 |
| Anticoagulant treatment (n, %) | 10(0.9) | 0.203 |
| ESWL history within 12-month (n, %) | 21(1.9) | ***0.082*** |
| RIRS history within 12-month (n, %) | 45(4.1) | 0.509 |
| PCNL history (n, %) | 40(3.7) | 0.407 |
| Incision lithotomy history (n, %) | 39(3.6) | 0.387 |
| Charlson comorbidity index |  | 0.857 |
| 0 | 878 (80.2) |  |
| 1 | 159 (14.5) |  |
| 2+ | 58 (5.3) |  |
| *Pre- and intraoperative characteristics* |  |  |
| Ureteralstent indwelling time (Days) | 20(14, 30) | 0.819 |
| Access sheath size (Fr) |  | ***0.037*** |
| 12/14 Fr (n, %) | 339(31.0) |  |
| 14/16 Fr (n, %) | 756(69.0) |  |
| Time in RIRS (mins) |  | ***0.052*** |
| Time in RIRS (only scope time) ≥30mins (n, %) | 382 (34.89) |  |
| Time in RIRS (only scope time) <30mins (n, %) | 713 (0.6511) |  |
| Time in extracting stone fragments (Mins, IQR) | 13(7, 23) | 0.249 |
| *Imaging information* |  |  |
| Number of stones |  | ***0.05*** |
| One (n, %) | 450(41.1) |  |
| Two (n, %) | 286(26.1) |  |
| Three (n, %) | 120(11.0) |  |
| Four (n, %) | 66(6.0) |  |
| Five and more than five (n, %) | 173(15.8) |  |
| Stone Location |  | 0.599 |
| Upper calyx (n, %) | 34(3.1) |  |
| Middle calyx (n, %) | 72(6.6) |  |
| Lower calyx (n, %) | 170(15.5) |  |
| Renal pelvis (n, %) | 165(15.1) |  |
| Ureteropelvic Joint (n, %) | 31(2.8) |  |
| Ureter (n, %) | 163(14.9) |  |
| Multiple locations (n, %) | 496(45.3) |  |
| Stone size (mm^3^, IQR) | 673.4(364, 1203) | 0.356 |
| Average CT value of stones (HU) | 1052.5(825, 1259) | 0.143 |
| *Laboratory tests* |  |  |
| Blood RBC count(10^12/L) | 4.64 (4.3, 5.01) | ***0.018*** |
| Plasma hemoglobin(g/L) | 140 (126,151) | ***0.001*** |
| Alkaline phosphatase (IU/L) | 75 (63, 90) | ***0.011*** |
| neutrophilic granulocyte segmented form in blood (%) | 3.65 (2.95, 4.44) | 0.898 |
| Percentage of neutrophilic granulocyte segmented form in blood(10^9/L) | 60 (54.4, 65) | 0.483 |
| Absolute value of blood monocyte (%) | 0.39 (0.31, 0.49) | 0.795 |
| Percentage of eosinophils in blood (%) | 3.1 (2.1, 4.5) | 0.302 |
| Absolute value of eosinophils in blood (10^9/L) | 0.19 (0.13, 0.28) | 0.411 |
| Percentage of basophils(%) | 0.5 (0.4, 0.7) | 0.302 |
| Absolute value of blood basophils (10^9/L) | 0.03 (0.02, 0.05) | ***0.013*** |
| Lymphocyte percentage (%) | 29.2 (24.4, 34.6) | 0.331 |
| Lymphocyte absolute value (10^9/L) | 1.79 (1.43, 2.17) | 0.130 |
| White blood cell count (10^9/L) | 6.13 (5.22, 7.33) | 0.723 |
| Blood Globulin (g/L) | 26.5 (23.9, 29.5) | ***<0.001*** |
| Albumin/Globulin ratio | 1.62 (1.45, 1.83) | ***<0.001*** |
| Prothrombin time (S) | 11.1 (10.7, 11.6) | 0.982 |
| Thrombin time (S) | 19.2(18.4, 20.2) | 0.736 |
| Thrombin time ratio | 1.18 (1.13, 1.24) | 0.474 |
| International standardized ratio | 0.96 (0.91, 1.01) | 0.464 |
| Blood Fibrinogen (g/L) | 2.91 (2.47, 3.37) | ***0.007*** |
| Platelet count (10^9/L) | 180 (139, 221) | 0.588 |
| Activated partial thromboplastin time ratio | 0.98 (0.89, 1.06) | 0.623 |
| Serum glucose (mmol/L) | 5 (4.64, 5.45) | 0.228 |
| Preoperative urine nitrite |  | ***<0.001*** |
| Positive (n, %) | 53 (4.8) |  |
| Negative (n, %) | 1095 (95.2) |  |
| Urine bacterial count (/uL) | 39 (17, 92) | ***0.015*** |
| Urine leukocyte count (/HP) | 21 (9, 46) | ***0.017*** |
| Urine purulent cells |  | ***0.026*** |
| Negative (n, %) | 1045 (95.4) |  |
| + (n, %) | 36 (3.3) |  |
| ++ (n, %) | 10 (0.9) |  |
| +++(n, %) | 4 (0.4) |  |
| Urine bacterial culture |  | ***<0.001*** |
| Positive (n, %) | 249 (22.7) |  |
| Negative (n, %) | 846 (77.3) |  |
| Urine RBC count | 1271 (110, 6015) | 0.969 |
| Urine glucose |  | 0.524 |
| Negative (n, %) | 1030 (94.1) |  |
| Positive (n, %) | 65 (5.9) |  |
| Triglyceride (mmol/L) | 1.67 (1, 2.03) | 0.595 |
| High density lipoprotein (mmol/L) | 1.23 (1.02, 1.38) | 0.379 |
| Low density lipoprotein (mmol/L) | 2.62 (2.19, 2.98) | 0.749 |

1: Time in RIRS (Mins) means only the flexible scope period of use

***:** Univariate analysis regression result obtained from univariate logistics regression.
